# The Informed Verdict

**Published:** 1918-03-09

**Authors:** 


### THE INFORMED VERDICT.

We have no lack of critics nowadays, and not a few of them are of the noisy order. Viewing them with all charity it is impossible to avoid the suspicion that some of them are at least as anxious to announce their individual activities as they are to reform the abuses which they condemn. Perhaps a more general comment would be that the critic so often limits his view to the particular point which strikes his attention. On the other side, and as a contrast, is the fault of wholesale and undiscriminating eulogy. Because certain arrangements have on the whole been successful and worked well the picture is presented in tints which imply that actual perfection has been reached. If the one side speaks as if individual blunders proved a general failure, the other treats a fair measure of success as a warrant of triumph in details. The independent observer will be wise to merge the two verdicts and to conclude that by listening to both voices he gets an approximation to the truth.

Now and again, fortunately, we are permitted the conclusions formed by an authority who has no personal ends to serve and who at the same time has had the opportunity of full information. Such a position may fairly be claimed for General Sir William Robertson when he speaks of the Military Medical Service. His tribute has high value, first because of the positions he has held, and, secondly, because it is a reasoned one. Without question the preservation of the health of our armies in the field has been a contribution of high value to the national cause, and the result is largely due to the application of practical medicine and scientific research. The medical profession may well be content with the thanks of the Army expressed in the words of one of our greatest soldiers. At the same time the profession will not be so foolish as to believe that perfection has been reached or that every Army doctor is a model of professional skill or a pattern of common sense and discretion. In the stress of things human weaknesses betray themselves. What we can justly say is that in the record of the R.A.M.C. such rare incidents have not hindered a large measure of success.

If Sir William Robertson is qualified to judge the Medical Service of the Army he has still a greater authority in reference to the fighting ranks. While he reminds us of what the Army owes to the medical profession he reminds us also of what the nation owes to the Army. Without "these men of ours" our fate would have been that now suffered widely in Eastern Europe, and shame it will be to us if our wounded and incapacitated soldiers are allowed to want what is necessary for their welfare. Are these experiences to be repeated in the next generation? A recent German writer has answered this question by telling us that wars are inevitable, and that peoples must be disciplined and trained in preparation for them. Our British soldier replies that only by full success now can the world be saved from that "disgrace to civilisation" which consists in the maintenance by nations of great armies for the purpose of destroying each other. A sharper contrast between two ideals can hardly be imagined, and the issue must go forward to settlement. It is presented on our side by one who knows the spirit both of soldiers and civilians, and we could not wish a better qualified spokesman.

